# Survival and mortality rates in early onset dementia

**DOI:** 10.1136/jnnp-2025-336805

**Published:** 2025-11-04

**Authors:** Kasper Katisko, Mikko Aaltonen, Kalle Aho, Sami Heikkinen, Ave Kivisild, Adolfina Lehtonen, Laura Leppänen, Iina Rinnankoski, Helmi Soppela, Laura Tervonen, Noora Maria Suhonen, Annakaisa Haapasalo, Päivi Hartikainen, Johanna Krüger, Eino Solje

**Affiliations:** 1 Institute of Clinical Medicine - Neurology, University of Eastern Finland, Kuopio, Finland; 2 Law School, University of Eastern Finland, Joensuu, Finland; 3 Research Unit of Clinical Medicine, Neurology, University of Oulu, Oulu, Finland; 4 MRC, Oulu University Hospital, Oulu, Finland; 5 Neurocenter, Neurology, Oulu University Hospital, Oulu, Finland; 6 A.I. Virtanen Institute for Molecular Sciences, University of Eastern Finland, Kuopio, Finland; 7 Neuro Center - Neurology, Kuopio University Hospital, Kuopio, Finland

**Keywords:** FRONTOTEMPORAL DEMENTIA, VASCULAR DEMENTIA, EPIDEMIOLOGY, ALZHEIMER'S DISEASE, Prognosis

## Abstract

**Background:**

The socioeconomic burden of early onset dementia (EOD) defined as disease onset before the age of 65 years, is substantial due to its widespread disabling effects in relatively young individuals. While dementia is widely recognised as a major contributor to mortality among the elderly, only a limited number of studies have assessed survival and factors associated with prognosis specifically in EOD.

**Methods:**

A population-based cohort study, encompassing all incident EOD cases from two defined regions in Finland. The survival and all-cause mortality rates in EOD and its subtypes were evaluated from January 2010 to December 2021. All visits at the dementia outpatient clinics were reviewed and manually re-assessed (n=12 490), resulting in 794 validated EOD cases of Alzheimer’s disease (AD), frontotemporal dementia (FTD), alpha-synucleinopathy (α-SYNU) and other EOD spectra. Region-, age- and sex-matched control groups without neurodegenerative diseases were created from nonselective general population data registers (1:10 case to control ratio, 7930 controls in total).

**Results:**

The median survival for EOD was 8.7 years, with the shortest survival in the FTD (6.9 years) and α-SYNU groups (7.0 years), followed by the AD group (9.9 years). Compared with controls, mortality was significantly higher in the total EOD group (HR=6.56, 95% CI=5.56–7.74, p<0.001). Among the dementia subtypes, FTD spectrum patients had the highest all-cause mortality risk compared with controls (HR=13.75, 95% CI=10.25–18.43, p<0.001). Male sex, older age, several comorbidities and lower level of education were associated with increased mortality, but these were not EOD-specific.

**Conclusion:**

EOD diagnosis significantly deteriorates patients’ survival, with significant variation between different diagnostic groups and in relation to patients’ demographic factors.

**WHAT IS ALREADY KNOWN ON THIS TOPIC**

- Epidemiological data on survival and mortality rates for specifically early onset dementia (EOD) is notably scarce, with substantial register-based methodological variability and limitations. Most recent meta-analysis on dementia survival warranted further epidemiological research to investigate the potential sources of divergence in mortality risks, especially in distinct dementia types.

**WHAT THIS STUDY ADDS**

- Our study provides up-to-date EOD survival rates in a validated population-based EOD cohort, and highlights the substantial effect caused by EOD diagnosis to patients’ mortality. Significant variations in mortality rates were observed in relation to specific EOD clinical subtypes, patients’ sex, comorbidities, age and family history. Although survival time in years is shorter in older dementia patients, the impact of dementia diagnosis to all-cause mortality is even more substantial in the EOD group when compared with the general population without dementia.

**HOW THIS STUDY MIGHT AFFECT RESEARCH, PRACTICE OR POLICY**

- Accurate up-to-date data on the survival and mortality rates of EOD are crucial in designing healthcare structures, comprehensive patient care and clinical trials.

## Introduction

 The number of people living with dementia is expected to rise globally,^1^ causing enormous socioeconomic challenges worldwide.^2^ Approximately 5% of dementia cases with an overall global prevalence estimate of 119 per 100 000 inhabitants are categorised as early (or young) onset dementia (EOD), characterised by disease onset before the age of 65 years.^3^ Due to the earlier disease onset, disability occurs in the working-aged population with less morbidity compared with individuals aged >65 years, emphasising the overall burden caused solely by EOD. Our recent epidemiological study indicated higher incidence rates of EOD than previously reported,^4^ further highlighting the importance of this specific dementia category.

A dementia diagnosis is considered to substantially affect patients’ life expectancy with significantly increased all-cause mortality rates (5.90 times larger HR).^5^ Survival times in patients with dementia vary markedly even in patients with the same diagnoses, with median survival times ranging from 3.3 to 11.7 years.^6^ Although survival rates of dementia in general have been widely studied, only a few studies have evaluated survival rates specifically in EOD patients let alone in all of its clinical subtypes.

It has been suggested that survival time is shorter in late-onset dementia compared with EOD, although EOD substantially increases mortality.^7^ In addition to onset age-related differences in survival, dementia type-specific differences have also been suggested with higher mortality rates found in non-Alzheimer’s disease dementias compared with Alzheimer’s disease (AD), although contrary results also exist.^5^

Notably, most previous studies have evaluated either survival in dementia in general (including all age groups), focused on late onset (>65 years) diseases or specifically to AD or unspecified dementia.^5^ On the other hand, study cohorts specifically considering EOD patients have been of limited cohort sizes, have not included all major dementia subtypes, or included a patient or control cohort not representing the overall population, or have not been able to evaluate other potential variables or comorbidities affecting the heterogeneity observed in survival rates.^57^,^10^

Accurate knowledge on survival and mortality rates of EOD and its subtypes is important not only to healthcare providers considering appropriate resource allocation and treatment strategies, but also to patients and caregivers for future care planning.

In our study we aimed to evaluate survival and mortality rates in a large and comprehensive EOD cohort including all major dementia subtypes. In addition, we evaluated potential factors associated with mortality in patients with EOD.

## Methods

### Study design and participants

In this cohort study with a population-based approach, we systematically identified all clinical diagnoses of EOD from two university hospital districts of the defined areas (provinces) of Northern Savonia (20 367 km^2^, 30–64 year-old inhabitant population: 106 579) and Northern Ostrobothnia (37 149 km^2^, 30–64 year-old inhabitant population: 174 249) in Finland. The methodology used to group the incident cohort was based on the reconstructed cohort design.^11^,^13^ The memory clinics of Kuopio University Hospital (KUH) in Northern Savonia and Oulu University Hospital (OUH) in Northern Ostrobothnia represent tertiary and primary regional referral centres of these areas for all citizens ≤65 years with neurodegenerative disease-related cognitive or other related complaints. Regardless of patients’ socioeconomic status or primary healthcare provider (public healthcare, occupational healthcare, private healthcare), all patients aged ≤65 years are referred to these defined centres for a diagnostic evaluation in corresponding geographical areas (ie, all EOD diagnoses in Finland are set exclusively in specific centres based on patients’ geographical residence).

All patients admitted to KUH and OUH Neurology outpatient clinics with a progressive neurodegenerative disease during 2010–2021 were identified from patient data registries (initial n=12 490). The clinical data of each potential patient including follow-up information were thoroughly re-evaluated and validated by experienced neurologists specialised in neurodegenerative diseases, to confirm the diagnoses based on the prevailing clinical criteria.^14^,^21^ Patients with a dementia diagnosis before or at the age of 65 years were included in the final cohort. To ensure the accurate classification of cases as EOD, the age at diagnosis was utilised as the inclusion threshold, rather than the estimated age at symptom onset. Patients without a progressive disease course as well as patients diagnosed with Down’s syndrome, dementia due to intellectual disability, alcohol-related and other secondary dementias were excluded from the study.

The eventual study cohort included 794 incident EOD cases: 421 cases with AD spectrum diagnosis, 180 cases with frontotemporal dementia (FTD) spectrum diagnosis, 46 cases with the alpha-synucleinopathy (α-SYNU) spectrum diagnosis and 147 cases with other EOD. The FTD group included 101 behavioural variant FTD, 25 primary progressive aphasia, 21 progressive supranuclear palsy, 8 corticobasal degeneration, and 25 FTD-ALS patients. The α-SYNU group included 31 patients with dementia with Lewy bodies (DLB) and 15 multiple system atrophy patients. The “Other EOD” group included 97 patients with vascular cognitive disorders, 22 combined AD+vascular dementia patients, 8 combined AD+DLB patients, 15 Huntington’s disease patients, and 5 unspecified progressive dementia cases. Patients classified into the mixed-dementia groups (AD+vascular or AD+DLB) had imaging-supported diagnoses that fulfilled the diagnostic criteria for both AD and VaD, or both AD and DLB, respectively.^19^,^21^ Incidence and prevalence rates of this cohort have been reported and characterised earlier.^4^

Along with basic demographic factors such as sex, age and level of education, we evaluated the role of comorbidities and familial history to the patients’ survival. We included the most common comorbidities that are likely to affect survival and divided them into four categories: (1) Cardiovascular comorbidities (ICD-10 codes I10–I79); (2) Diabetes (ICD–10 codes E10–E14); (3) Malignant neoplasms/cancers (ICD–10 codes (C00–C97+D00–D09); and (4) Chronic pulmonary diseases (ICD–10 codes J40–J47). Comorbidities were collected from the Finnish Care Register for healthcare and included from a 10 year time period before the EOD diagnosis of each patient. As for family history, we collected each patients’ familial history for dementia categorised as 1, 2, 3, 3.5 or 4, using a modified Goldman score^22 23^ as a guideline also in other groups than FTD. We also collected data on familial psychiatric history, including any reported psychiatric disease in the first- or second-degree relatives of the patients. Educational level of each patient was obtained from Statistics Finland national registers. Educational level was categorised into three categories: (1) Primary/basic level (incl. ISCED–11 levels 0–2); (2) Secondary level (incl. ISCED–11 levels 3–4); and (3) Tertiary level (incl. ISCED–11 levels 5–8).^24^

Using a personal social security number, each patient is linked to national registers of the Finnish Social and Health Data Permit Authority. These registers provide comprehensive lifetime statistical information, including education history from Statistics Finland national register and mortality data, which is updated daily, with times of deaths from Population Register Centre of Finland.

### Standard protocol approvals, registrations and patient consents

The protocol was registered to ClinicalTrials.gov (NTC06209515). As a non-interventional registry study, no additional data were collected directly from the study individuals. According to the Finnish legislation (552/2019), no consents from the study individuals were required for retrospective studies. Thus, an independent Ethical Committee evaluation was not required for this study. The research protocol was approved by the Finnish Social and Health Data Permit Authority Findata (THL/2841/14.02.00/2022).

### Statistical analysis

We used Kaplan-Meier estimates to plot basic mortality and survival patterns over the follow-up and calculated both 25% and median survival times (from diagnosis to death). Cox proportional hazard regression analysis was used to examine factors associated with mortality. We fitted models comparing cases to healthy controls, and also models examining factors associated with mortality among all EOD patients and in each of the four diagnostic groups. All analyses were conducted using Stata 18.

## Results

The study cohort including demographics of survival and mortality rates of each diagnostic group is described in table 1.

**Table 1:** Characteristics of the EOD cohort (N=794) with survival and mortality in each diagnostic group

	All EOD (n=794)	AD spectrum (n=421)	FTD spectrum (n=180)	Alpha-synucleinopathies (n=46)	Other EOD* (n=147)
Age at diagnosis (years, mean)	59.6	60.1	58.8	60.5	58.9
Sex (n, M/F)	393/401	175/246	96/84	33/13	89/58
Educational level basic/secondary/tertiary †, %	23/49/28	22/47/31	18/51/31	26/48/26	29/54/17
Family history of dementia (%)‡	28.3%	33.3%	22.8%	23.9%	21.9%
Comorbidity (%)					
Cardiovascular	35.0%	27.3%	31.7%	47.8%	57.1%
Diabetes	9.3%	6.2%	7.2%	13.0%	19.7%
Cancer	6.9%	5.2%	4.4%	15.2%	12.2%
Pulmonary	6.9%	5.9%	5.6%	13.0%	9.5%
Observed deaths during the study period, n (%)	215 (27.1%)	78 (18.5%)	88 (48.9%)	18 (39.3%)	31 (21.1%)
Survival time from diagnosis (years, median)	8.7	9.9	6.9	7.0	>10.0§
Survival time from diagnosis (years, 25%)	5.6	6.9	3.2	3.6	6.5
Mortality rate vs controls¶, H (95% CI)	HR=6.56 (5.56–7.74)	HR=4.62 (3.55–6.01)	HR=13.75 (10.25–18.43)	HR=7.85 (4.37–14.10)	HR=4.39 (2.91–6.64)

* Other EOD group includes vascular cognitive disorders (n=97), Alzheimer’s disease combined with dementia with vascular dementia (n=22), Alzheimer’s disease combined with dementia with Lewy bodies (n=8), Huntington’s disease (n=15), and undefined EOD without a specific diagnosis (n=5).

† Educational levels categorised as basic education=1 (ISCED-11 categories 0–2), secondary education=2 (ISCED-11 categories 3–4), and tertiary education=3 (ISCED-11 categories 5–8).

‡ Family history considered positive with Goldman scores=1–3, see methods section for details.

§ Exact median survival was not available due to median survival extending the range of study period.

¶ Each diagnostic group had separate region- age- and sex matched control group without neurodegenerative disorders, with 1:10 case to control ratio.

AD, Alzheimer’s disease; EOD, early onset dementia; F, female; FTD, frontotemporal dementia; M, male.

During the study period, 215 out of 794 (27.1%) patients died. The median survival time from EOD diagnosis to death was 8.7 years in the total EOD group. In the separate dementia subtype groups, the shortest median survival time was observed in the FTD group (6.9 years), followed by the α-SYNU group (7.0 years), and the AD group (9.9 years). Within the clinical subgroups, FTD-ALS patients had the shortest survival time (2.5 years). Survival was, however, still short in the FTD group even when the FTD-ALS patients were excluded (7.2 years). In the Other EOD group, the Kaplan-Meier survival curve did not decrease below 0.5 during the follow-up period. Thus, the exact median survival could not be calculated without additional assumptions (median survival was at least >10 years). For this reason, we also calculated 25% survival times, indicating the time by which 25% of the cases had died. The 25% survival times were 3.2 years in FTD, 3.6 years in α-SYNU, 6.9 years in AD and 6.5 years in the Other EOD group, giving further proof of high mortality risks especially in the FTD and α-SYNU groups. Vascular cognitive disorder was the most common diagnosis in the Other EOD group and thus was evaluated also separately. The median survival was not available for these patients due to same reason as in the Other EOD group in general (median survival >10 years). The 25% survival time was 6.7 years in the vascular cognitive disorder group.

Compared to region, age, and sex matched controls, risk of all-cause mortality was significantly higher in the total EOD group (HR=6.56, 95% CI=5.56–7.74, p<0.001). FTD spectrum patients had the highest all-cause mortality risk compared with their controls (HR=13.75, 95% CI=10.25–18.43, p<0.001), followed by α-SYNU vs controls (HR=7.85, 95% CI=4.37–14.10, p<0.001), AD vs controls (HR=4.62, 95% CI=3.55–6.01, p<0.001), and Other EOD group vs controls (HR=4.39, 95% CI=2.91–6.64, p<0.001). From the Other EOD group, patients with vascular cognitive disorders were further evaluated separately, showing increased mortality compared with their matched controls (HR=4.66, 95% CI=2.70–8.04, p<0.001) (table 1).

Between the diagnostic groups, age, sex, comorbidity and education adjusted mortality rates were significantly higher especially in the FTD vs AD group (HR=3.02, 95% CI=2.21–4.13, p<0.001) and in the α-SYNU group vs AD (HR=2.68, 95% CI=1.58–4.55, p<0.001). There was no significant difference between the Other EOD group vs AD group (HR=1.05, 95% CI=0.68–1.62, p=0.836).

Although age, sex, all four comorbidity categories and education level were significant independent factors in the regression models used to calculate the hazard ratios for mortality (EOD vs controls; individual disease groups vs controls), including these as covariates did not show any notable effect on the observed results or the above reported hazard ratios.

**Figure 1:**
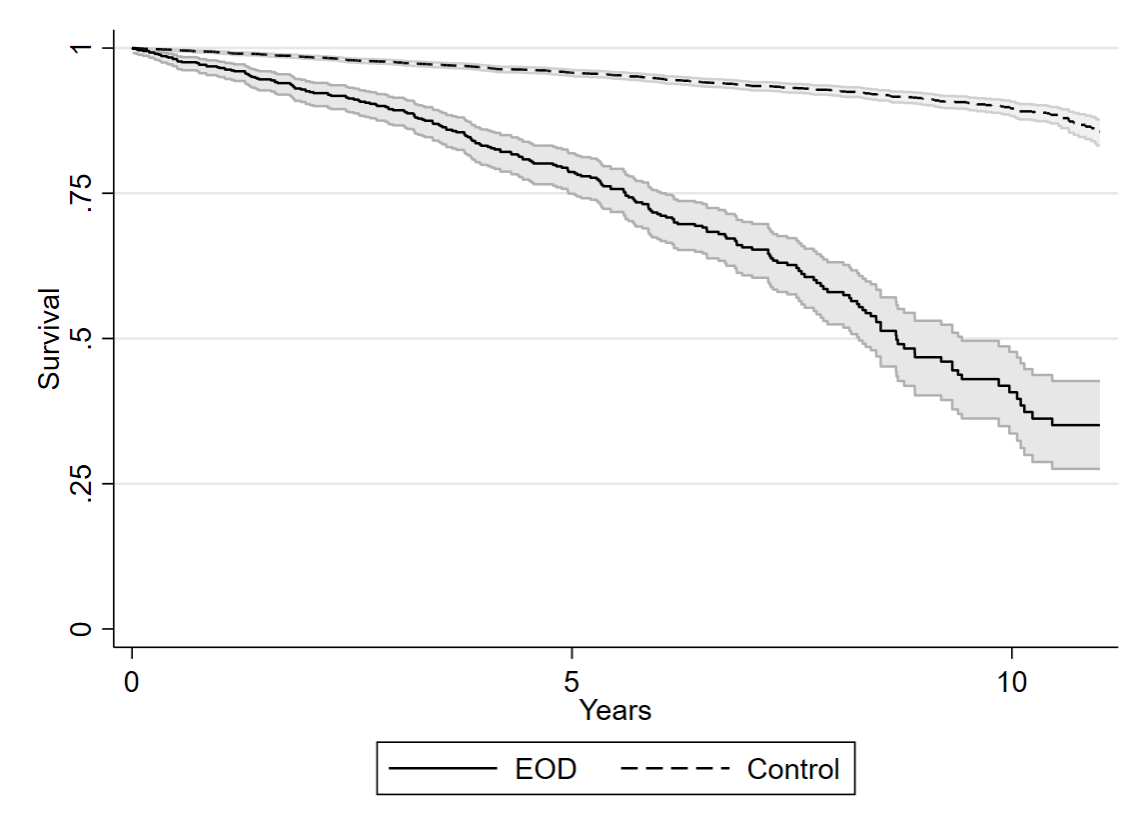
Survival estimates for early onset dementia (EOD) vs controls. Kaplan-Meier survival estimates with 95% CIs for EOD patients compared with region, age, and sex-matched controls. The median survival time from EOD diagnosis to death was 8.7 years in the total EOD group. Risk of all-cause mortality was significantly higher in the total EOD group (HR=6.56, 95% CI=5.56–7.74, p<0.001) vs controls.

**Figure 2:**
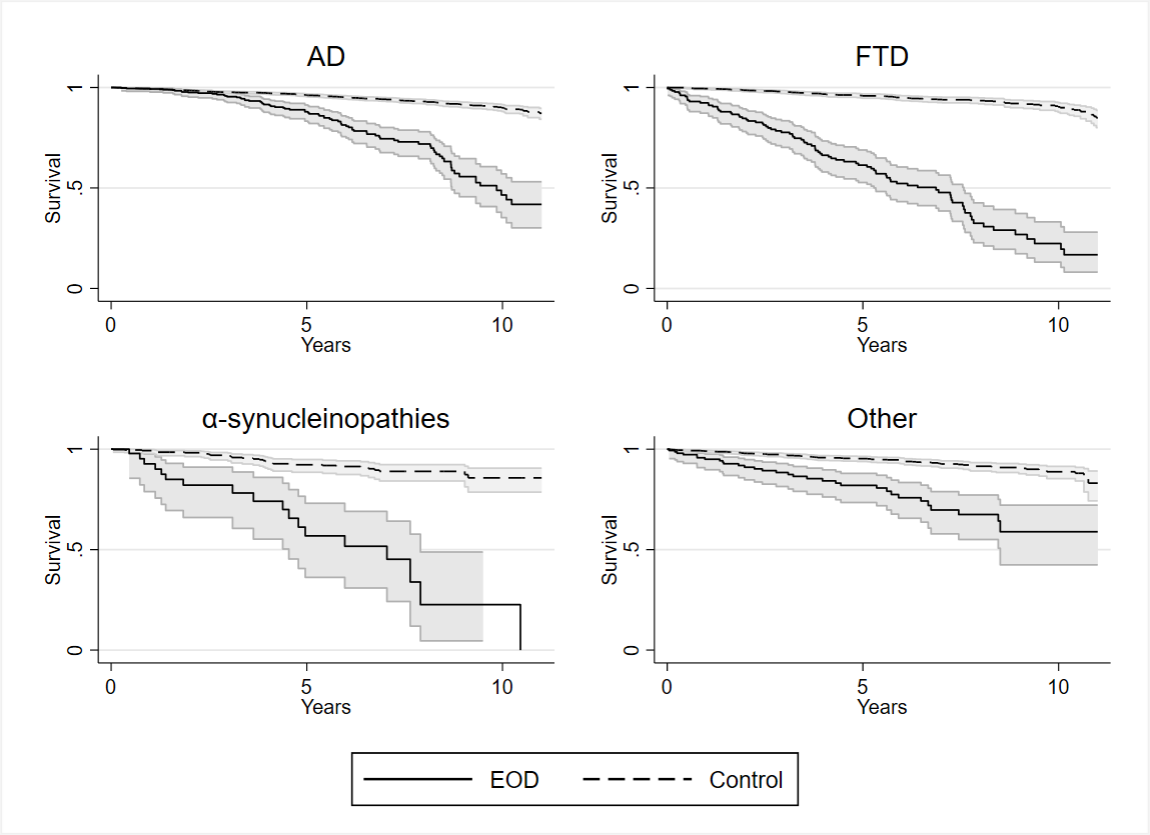
Survival estimates separately for early onset Alzheimer’s disease, frontotemporal dementia, alpha-synucleinopathies and other early onset dementia (EOD) *vs* controls. Kaplan-Meier survival estimates with 95% CIs in early onset Alzheimer’s disease (AD), frontotemporal dementia (FTD), alpha-synucleinopathies (α-SYNU) and other early onset dementia (Other) compared with disease group specific region, age, and sex-matched controls. The other EOD group includes vascular cognitive disorders (n=97), Alzheimer’s disease combined with dementia with vascular dementia (n=22), Alzheimer’s disease combined with dementia with Lewy bodies (n=8), Huntington’s disease (n=15), and undefined EOD without specific diagnosis (n=5). All-cause mortality rates in each group vs controls: FTD vs controls: HR=13.75, 95% CI=10.25–18.43, p<0.001; α-SYNU vs controls: HR=7.85, 95% CI=4.37–14.10, p<0.001; AD vs controls: HR=4.62, 95% CI=3.55–6.01, p<0.001; and other EOD vs controls: HR=4.39, 95% CI=2.91–6.64, p<0.001.

Kaplan-Meier survival estimates for the total EOD cohort vs controls are shown in figure 1, and survival estimates of the separate clinical subgroups vs their matched controls are shown in figure 2.

In the regression analysis including the total EOD group and controls, higher level of education associated with reduced mortality risk (second degree vs primary education HR=0.81, 95% CI=0.68–0.97, p=0.022 and third degree vs primary education HR=0.57, 95% CI=0.46–0.72, p<0.001), whereas higher age at diagnosis (HR=1.06, 95% CI=1.04–1.08, p<0.001), male sex (HR=1.83, 95% CI=1.55–2.15, p<0.001), cardiovascular comorbidities (HR=1.42, 95% CI=1.19–1.69, p<0.001), history of diabetes (HR=1.97, 95% CI=1.56–2.48=, p<0.001), history of cancer (HR=1.95, 95% CI=1.54–2.47, p<0.001), and history of pulmonary diseases (HR=1.45, 95% CI=1.11–1.90, p=0.006) associated with an increased mortality risk.

When evaluating only the EOD group (without controls in the model), independent significant factors associating with increased mortality were FTD diagnosis (HR=3.16, 95% CI=2.31–4.32, p<0.001), α-SYNU diagnosis (HR=2.77, 95% CI=1.63–4.69, p<0.001), male sex (HR=1.59, 95% CI=1.20–2.09, p=0.001), higher age at diagnosis (HR=1.03, 95% CI=1.01–1.06, p=0.019), Goldman category 1 (HR=2.19, 95% CI=1.03–4.67, p=0.042), and a history of diabetes (HR=1.69, 95% CI=1.12–2.54, p=0.012). Higher educational level (third degree vs primary level) showed a trend towards decreased mortality also separately in EOD patients, but the HR was not significant (HR=0.76, 95% CI=0.51–1.14, p=0.157). Family history of psychiatric disorders, other Goldman categories, or other comorbidity categories did not associate with increased or decreased mortality rates in the EOD patients. All of the evaluated factors and their association with mortality in EOD are summarised in table 2.

**Table 2:** Summary table for the evaluated variables and their independent association with mortality in EOD (N=794)

	Association or effect on mortality in EOD, ↑, ↓ or – with HR and 95% CIs
EOD subtype (FTD or α-SYNU vs AD or other EOD)	↑↑↑ for FTD (HR=3.16, 2.31 to 4.32), ↑↑ for α-SYNU (HR=2.77, 1.63 to 4.69)
Male sex	↑ (HR=1.59, 1.20 to 2.09)
Older age at diagnosis	↑ (HR=1.03, 1.01 to 1.06)
Comorbidities (diabetes)	↑ (HR=1.69, 1.12 to 2.54)
Comorbidities (cardiovascular)	- (HR=1.00, 0.73 to 1.35)
Comorbidities (cancers)	- (HR=1.21, 0.71 to 2.04)
Comorbidities (pulmonary)	- (HR=1.04, 0.59 to 1.83)
Family history of dementia (autosomal dominant, Goldman category 1)	↑↑ (HR=2.19, 1.03 to 4.67)
Psychiatric family history	- (HR=1.32, 0.56 to 3.10)
Higher educational level	- (↓)* (HR=0.76, 0.51 to 1.14)

“↑” indicates significantly increased mortality and shorter survival. “↓” indicates significantly decreased mortality and longer survival. “-“ Indicates no significant association. Number of ↑ demonstrates the size of the observed HR (↑ = HR > 1.0, ↑↑ = HR > 2.0, ↑↑↑ = HR > 3.0).

* Non-significant trend for decreased mortality in tertiary level education vs primary level education.

AD, Alzheimer's disease; EOD, early onset dementia; FTD, frontotemporal dementia; α-SYNU, Alpha-synucleinopathies.

Out of the 180 FTD patients, 42 (23.3%) carried the *C9orf72* hexanucleotide repeat expansion (HRE), the most common genetic cause of FTD (*C9orf72* HRE status was available for 85 patients). The *C9orf72* HRE did not associate with increased or decreased mortality rate compared with the *C9orf72* HRE non-carriers (HR=1.35, 95% CI=0.70–2.62, p=0.366). Data for other causal genetic mutations were not available for this cohort.

The survival of the patients remained relatively stable during the 12 year study period (apart from random variation between separate single years), indicating no systematic changes in the EOD prognosis from 2010 to 2022.

## Discussion

Here, we have reported up-to-date survival and mortality rates in a large and carefully validated, population-based EOD cohort. Mortality rates were significantly affected by the specific EOD diagnosis, with FTD and α-SYNU groups having the shortest survival. Male sex, older age, several comorbidities, and lower level of education associated with increased mortality, but these were not specific to EOD (ie, associations were equal or even stronger in controls). Autosomal dominant family history associated with increased mortality, whereas psychiatric family history did not.

In relation to the devastating impact caused by particularly EOD to the patients, caregivers, and society, surprisingly few studies have evaluated the survival and prognostic factors specifically in EOD. A relatively small prospective study from the Netherlands with 198 EOD cases and 77 deaths reported a 9.3 year median survival from EOD diagnosis to death in the total group, with AD patients having the shortest median survival time (8.6 years).^9^ Another study from a tertiary memory centre in Amsterdam reported 6.9 year median survival in EOD patients overall, with shortest survivals observed in patients with DLB (5.7 years) or “other dementia” (4.5 years) compared with AD or FTD (7.0 years in both groups).^7^ The median survival observed in our present study (8.7 years) settled in between the two previous studies, with FTD and α-SYNU groups having shorter survival times than the AD group. A meta-analysis from 2021 studying mortality rates in dementia including all age groups (EOD was not evaluated separately), reported shorter survival in non-Alzheimer’s dementia (all types grouped together) compared with Alzheimer’s dementia, with DLB patients having the shortest life expectancy.^5^ The consensus from these studies, including our present one, indicates shorter survival times in early onset FTD or α-SYNU patients compared with early onset AD. Notably, differences in the clinical subtypes considering the AD, FTD, and α-SYNU groups limits the comparability of the studies, although the cohorts included relatively similar patient populations. For example, motor neuron disease accompanied with FTD (FTD-ALS/MND) and extrapyramidal phenotypes are known to significantly decrease survival time in patients of the FTD spectrum.^25^ This was detected also in our cohort, although the survival time in the FTD group was short even after excluding the FTD-ALS patients.

We found higher level of education as a protective factor against mortality especially in the total cohort combining controls and EOD patients. The trend remained similar in EOD patients only (controls excluded) considering the hazard ratios, but without a statistical significance. This could be due to decreased statistical power in the EOD cohort alone compared with the total cohort. On the other hand, it is possible that education plays a more significant protective role in unscreened control populations compared with patients with progressive dementia, in whom the dementia itself generates a remarkable risk effect on mortality. The role of education in dementia risk or dementia survival in general is multifactorial. In particular, the ‘Cognitive Reserve Hypothesis’ has been suggested as the reason underlying reduced dementia risk in patients with higher education, but it might also explain why patients with higher education may conversely have more rapid disease progression when dementia does occur.^26^ Higher education is a well-known protective factor against dementia,^27^ whereas the association between the education level and survival has been found more contradictory.^26 28^ Importantly, although higher education may be associated with faster cognitive decline after disease onset, as proposed by the cognitive reserve hypothesis, it does not appear to affect overall survival or age at death, as suggested by both the present study and prior research.^26 29 30^ Despite experiencing more rapid cognitive decline, individuals with greater initial cognitive reserve continue to demonstrate better cognitive performance than those with lower reserve even 2 years after disease onset.^30^ This study was limited to evaluating survival and mortality outcomes and did not assess disease progression based on cognitive decline rates. Moreover, in addition to educational attainment, other contributors to cognitive reserve, such as engagement in leisure and physical activities later in life, may influence these associations^30^; however, such data were not available for analysis in the present cohort.

The comorbidities included in our analyses associated with increased mortality in the study cohort, but these effects were more evident also when controls without dementia diagnosis were included. Furthermore, comorbidity adjustments did not significantly affect the observed strong associations between specific dementia types and survival, indicating a robust independent effect caused by the EOD itself. When evaluating EOD patients separately, only history of diabetes showed independent significant association to increased mortality. Diabetes is a known risk factor for dementia, and pre-existing diabetes has previously been associated with shorter survival/increased mortality in EOD and late onset dementia (LOD) patients,^10 31^ supporting our present finding.

The effect of family history, including neurodegenerative and psychiatric diseases, on the EOD prognosis has not been systematically studied before. Positive family history of psychiatric diseases did not affect survival in our EOD patients. We found no previous studies evaluating this specific issue. Considering family history of dementia, we found that specifically Goldman score 1 indicating autosomal dominant inheritance associated with increased mortality rate, whereas *C9orf72* HRE status did not. In studies with AD patients, survival of the autosomal dominant patients has not been significantly different from the sporadic patients or the effect has been minor.^32 33^ Similarly, in FTD patients, Goldman score status did not significantly affect mortality or care home admission hazard rates.^25^ Thus, further studies are needed to examine these factors.

We also observed that higher age at diagnosis and male sex were independent factors affecting mortality in EOD. The effect of EOD diagnosis to all-cause mortality was substantial (6.6 HR), being slightly higher than the 5.9 HR reported in a large meta-analysis combining all dementia types and all age groups (ie, significantly older patients on average).^5^ Overall, although the survival time in years appears shorter in older patients, the impact of dementia diagnosis to all-cause mortality is more significant in the EOD age group than in the late onset patients when compared with the general population.^8 10^ Male sex increased mortality in the EOD patients in the present study, but the effect appeared similar in the EOD patients and in the control population. It is known that in the general population, on average, women live longer than men.^34^ Thus, the observed association between male sex and decreased survival in the EOD patients may not be related to the EOD diagnosis itself.

The limitations of the present study include the limited number of neuropathologically confirmed or definite diagnoses, as the majority of the patients in our cohort had a clinical diagnosis without genetic or neuropathological confirmation (42 of the FTD patients were definite due to *C9orf72* HRE). On the other hand, the diagnoses were made in tertiary memory centres by experienced neurologists using thorough clinical evaluations (comprehensive neuropsychological assessments, MRI and/or PET imaging, usually AD CSF biomarkers), and the diagnoses were further confirmed in our retrospective cohort validation phase. Of the AD cases, 62.2% had a positive CSF-AD biomarker profile.^4^ Of those not having β-amyloid and/or τ markers assessed (37.8%), a high number had a diagnosis supported by FDG-PET. Another limitation was the lack of statistical power regarding specific subgroup analyses, in which group sizes were small. We aimed to emphasise the cohort quality and accuracy rather than maximise the cohort size. The healthcare structure in Finland is exceptionally well suited for a population-based approach considering EOD epidemiology, as practically all EOD cases regardless of the initial healthcare provider or socio-economic status are referred to the university hospital of that geographical area. Finnish Administrative Registers have been validated and confirmed to provide accurate and good quality data, well suited for epidemiological research.^35 36^ Thus, we were also able to include a sufficient number of matched controls representing the general population in the same geographical areas and evaluate several demographic factors and comorbidities potentially affecting patient survival in EOD.

## Conclusion

In conclusion, our study provides up-to-date survival rates in EOD, and highlights the substantial effect caused by EOD diagnosis to patients’ mortality. Significant variations in mortality rates were observed in relation to specific EOD clinical subtypes, patients’ sex, comorbidities (especially history of diabetes), age and autosomal dominant family history.

## Data Availability

No data are available.
